# The Fiji Sugar Industry: Sustainability Challenges and the Way Forward

**DOI:** 10.1007/s12355-022-01132-4

**Published:** 2022-04-12

**Authors:** Mohseen Riaz Ud Dean

**Affiliations:** Faculty of Arts and Social Sciences, Anthropology Program, University of Waikato, Hamilton, New Zealand

**Keywords:** Crop, Farmers, Fiji, Sugarcane Production, Perfect Storm, Problems

## Abstract

This paper examines the problems facing the sugar industry in Fiji. It expands on the difficulties of world trade and the macro- and micro-problems that affects the sugar industry sternly. It also discusses local challenges associated with sugarcane crop production and sugar manufacturing in the country. Additionally, it provides an overview of some of the specific issues directly facing smallholder sugarcane growers. This study is based on a customised mixed-methods research conducted in the year 2015 in the sugarcane-producing areas on the islands of Viti Levu and Vanua Levu in Fiji. The 33 farmers and members of their households in the study comprised both descendants of the *Girmitiya* community brought from India under the colonial rule and the native *iTaukei* sugarcane farmers, who rely primarily on cash incomes derived from the sale of sugarcane and other diversified farm products. This paper demonstrates that the sugar industry in Fiji is currently facing a ‘perfect storm’—wave after wave of major difficulties coming all at once—and the country is beginning to tackle these problems only as they reach crisis point.

## Introduction

In the literature, there exists much information on the lives of the *Girmitiyas*—the people and their descendants whom the British brought between 1879 and 1916 from colonial India to work as indentured labourers on the sugarcane plantations of Fiji (Ali 1980; Lal 1992, 1997, 2004, 2011; Prasad 2004). Scholars have also studied the history of the *Girmitiya* indenture system in Fiji and the harsh condition of *Girmitiya* life under the British and Colonial Sugar Refinery (CSR) rule (1879–1973) (Subramani 1979; Carswell 2000, 2003; Prakash 2009). Similarly, much has already been written on the economics of sugarcane production (Reddy 2003a; Reddy 2003b; Mahadevan 2008; Kumari and Nakano 2016) and land tenure and its implications for the sugar industry in Fiji (Moynagh 1978; Kurer 2001; Lal et al. 2001).

However, there remains a vast opportunity to investigate and document both the contemporary problems and the unresolved traditional issues of the sugar industry in Fiji. Moreover, the impacts of the ongoing COVID-19 pandemic, although an issue of global significance, locally present an additional problem to the already multitude of issues that trouble the sugar industry in Fiji. Generally, the pandemic presents the people of Fiji, particularly the sugarcane farmers, with problems of livelihood security (Dean 2020). A recent paper by Sachan and Krishna (2021) focusing on the effects of the COVID-19 pandemic on the Fiji sugar industry has tried to fill some gaps in the literature. However, problems of the Fiji sugar industry remain underexplored in the academic discourse. This study, therefore, aims to fill these gaps.

The sugar industry in Fiji formally began in 1882, and by 1883, the cultivation of sugarcane in the country had displaced copra as the chief export crop (Dean 2019). The production of sugarcane eventually became the backbone of the country, and by the mid-1970s, Fiji was exporting raw sugar to the United Kingdom (UK) and the European Union (EU) (at that time, the European Economic Community). For more than a century since the industry’s inception, sugar remained the economic strength of the Fijian economy and the country’s development (Vaniqi 2012). However, beginning 1980s, the industry started to experience many difficulties.

One of the many challenges coalesced around lower crop productions. Lower crop production means lower sugar yields. For example, in 1996, the industry produced 437,921 tonnes of sugar. However, by 1998 the sugar yield decreased by almost 100,000 tonnes, as the industry only managed to produce 364,000 tonnes of raw sugar, earning the country US$122.9 million and generating 30% of Fiji’s agricultural gross domestic product (GDP) (Advameg 2016). Also, by the new millennium, the tourism sector had taken over from sugar as Fiji’s primary export industry. In addition, in the last decade, the average cost per tonne of sugar produced by Fiji’s four sugar processing mills stood at approximately FJ$262.50. It is well above the cost of production in most mills in India, for example FJ$70.00 per tonne. Fiji’s cost of production has also surpassed most of her peers in the African, Caribbean, and Pacific (ACP) group of countries, standing at number seven (Sharma 2005).

Today, the sugar industry is beset by an array of problems from both inside and outside the country. Outside the country, one of the significant problems coalesces around international sugar markets. As part of the EU, the UK was the biggest importer of raw sugar and was obliged to purchase sugar from Fiji tariff-free under Economic Partnership Agreements. However, in 2017, the EU terminated the preferential sugar access making the EU sugar market competitive. This decision of the EU made the ACP member countries (including Fiji), which have been enjoying free sugar quota access to the UK, compete globally for markets for their sugar production. Brexit has further aggravated the issue. In addition, Fiji lost over FJ$350 million in EU grants to the sugar industry over the 2006–2014 period because of the military coup of December 2006.

Added to this are the pending unresolved traditional issues such as the expiry of some 20,000 leases on native land in the sugarcane-producing areas, which has given rise to poverty and household food security implications, majorly for smallholder sugarcane farmers. In Fiji, most sugarcane cultivation takes place on lands leased to the farmers by the native *iTaukei* landowners. Decreasing profits from sugarcane production, coupled with cultivation, harvesting, and transportation costs and the non-renewal of land leases, have all led to the loss of farmer faith in the industry. Besides, the sudden increase in fertiliser prices and the purchasing of substitutes for soil fertilisation continue to affect farmer incomes.

As a result, many sugarcane farmers have moved to urban centres of Fiji and overseas to seek easier and better-paying jobs over the past twenty years. In the current times, most of the sugarcane fields are cultivated by the middle- to late-age farmers, with their children leaving to attend universities to receive the education they need for white-collar jobs. Consequently, the farmers of this middle–late-age group do not feel secure and are not passing on their farms to the next generations. Considering the sugar industry’s unresolved problems, particularly those related to the sugarcane farmers, the industry is now in crisis.

This study describes the problems facing the sugar industry in Fiji, many of which the country is only beginning to address as they reach crisis point. Of these problems, loss of farmer confidence and demoralisation are the most urgent, affecting sugarcane crop production, the overall objectives for the rejuvenation of the industry, including the country’s national goals. In addition, social contention and political conflict within the industry also continue to adversely affect the quarter of the country’s populations, the farmers, and members of their households, who depend in many ways, both directly and indirectly, on the sugar industry for their livelihoods.

## Methodology

The research methodological framework applied to this study builds on Carswell’s 1996–1997 ethnographical methodological framework (2000, 2003), whereby she studied the role of gender and familial relations to produce sugarcane crops in Fiji. Carswell’s study was limited to 20 smallholder sugarcane farmer households on the second main island, Vanua Levu, Fiji. However, the current study extends the scope to include smallholder sugarcane farmers and members of their households on both the main islands of Fiji: the western (Viti Levu) and the Northern (Vanua Levu). In addition, the study involves Charles and Fen (2007) approach of stratified convenience sampling for accessing and drawing a total of 116 research participants, including 29 smallholder and 4 large-scale farmers as key research participants, who were easily reachable and were willing to participate in the study (Table 1). The study obtained University of Waikato’s Faculty of Arts and Social Sciences Human Research Ethics Committee approval.

**Table 1:** Summary of research approach

Research techniques	Sample no	Research cohort type
Semi-structured interview	12	Government and the stakeholder institutions in the Sugar Industry: Ministry of Sugar (MoS), Ministry of Agriculture (MoA), Cane Producer Association (CPAs), Sugar Cane Growers Fund (SCGF), Sugar Cane Growers Council (SCGC), Fiji Sugar Corporation (FSC), FairTrade (FT), Sugar Research Institute of Fiji (SRIF), Sugar Research Group, Secretariat of the Pacific Community (SPC)/ EU
Focus group discussion	55	Smallholder sugarcane farmers, Sector officers, FT officer, Sugarcane cutters, Sugarcane lorry drivers, Sirdar, Paniwala, Hukmaan, Sugarcane producer association director, FSC field officer, family and relatives of sugarcane farmers
Participant observation Livelihood survey Informal farmer interviews Agroecosystem analysis	33 + 16	Master sugarcane farmers (descendants of Girmitiya and iTaukei) + Children, Grand children, and relatives of master farmers
Total	116	

**Fig. 1:**
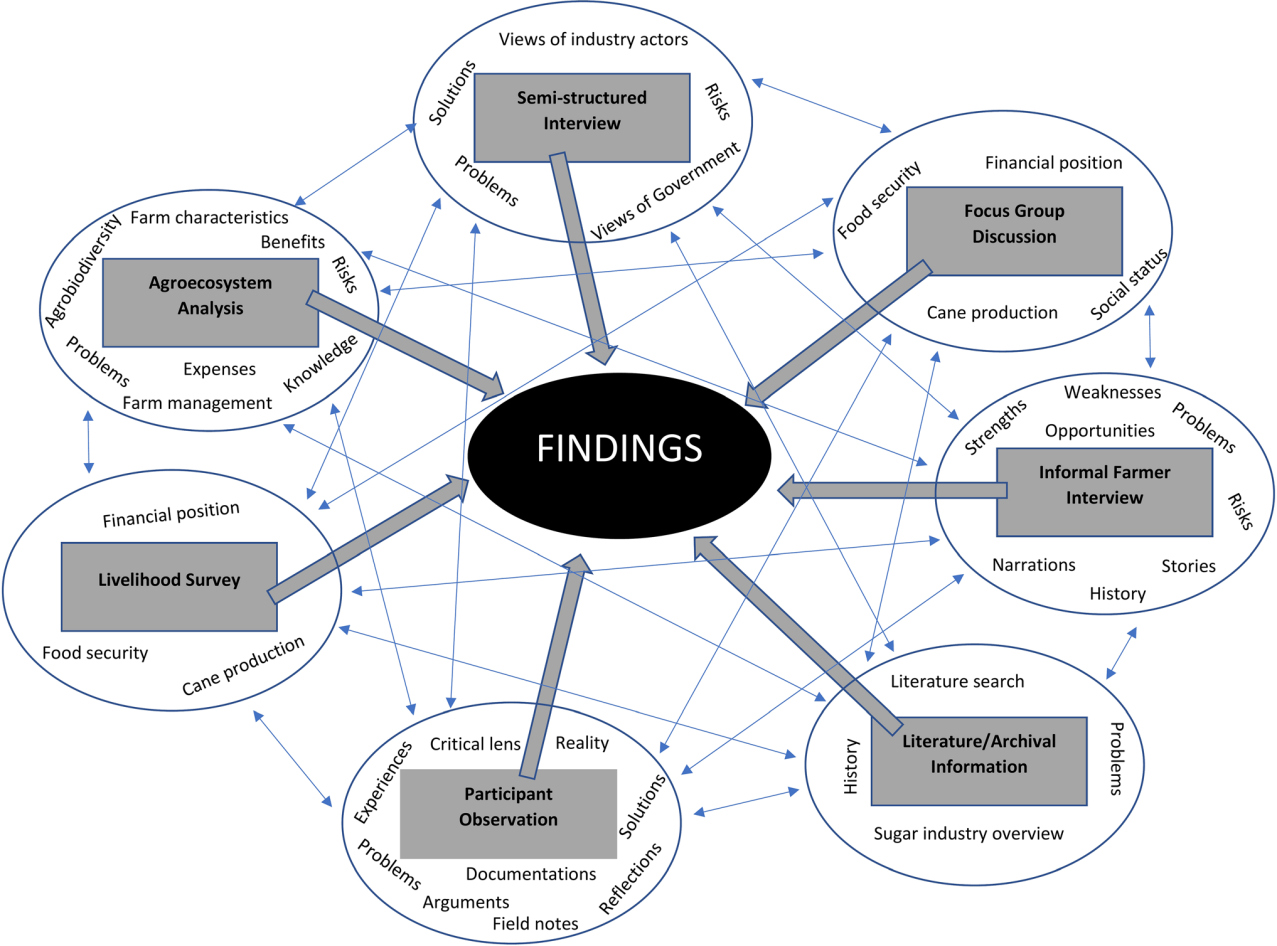
A customised mixed methodological framework

The study utilised a customised mixed methodological framework of qualitative and quantitative methods to generate insights (Fig. 1). The mixed-methods framework incorporated multiple research methods drawn from the disciplines of both social sciences (participant observation, semi-structured interviews, focus group discussions, informal interviews, and literature and archival research) and agronomy (agroecosystems analysis and livelihood survey). The customised framework helped in the triangulation of the findings generated. In addition, the strength of one method compensated for the weakness of the other method within the framework, making the data generated more robust, significant, and meaningful.

The qualitative aspects of the study involved the ethnographic method of participant observation that entailed observing and participating in the daily lives of the 33 master sugarcane farmers and members of their households. Taped semi-structured interviews were also conducted with critical respondents from other sugar industry stakeholders at the local and national levels, such as governmental representatives of the Ministry of Sugar (MoS) and Ministry of Agriculture (MoA), the employees of the Fiji Sugar Corporation (FSC), Sugar Research Institute of Fiji (SRIF), Cane Producer Association (CPAs), Sugar Cane Growers Fund (SCGF), and the Sugar Cane Growers Council (SCGC), among others. A total of 11 focus group discussions were also conducted as part of the study to gather the opinions and views of the farmers on the sugar industry crisis, the macro- and micro-problems of the sugar industry and to stimulate discussions concerning strategies for food and income security, and sustainable production of sugarcane crops.

Other qualitative methods included informal farmer interviews administered with the smallholder sugarcane farmers over casual settings such as during their lunch hours which helped to have deeper conversations around their technical knowledge of sugarcane farming. Field notes were also taken daily to record observations and experiences. In addition, photographs and video recordings of farming life and activities on the farm provided a valuable record for future reference. The literature from governmental and non-governmental agencies, the media, and academia was also consulted.

Quantitative information using methods of agroecosystem analysis and livelihood survey was obtained from farmer households. The agroecosystem analysis and livelihood survey complemented and supplemented the data obtained via the qualitative methods of inquiry. The livelihood survey combined the livelihood portfolio of rural activities and expenditures. It elicited accurate and detailed information and demonstrated and influenced the understanding of rural sugarcane farmer livelihoods. These quantitative methods also helped identify traditional agroecological approaches for sugarcane production, stability, sustainability, and equitability.

It is beyond the scope of this study to include all the other findings; however, the focus will be on the traditional unresolved and the current problems facing the sugar industry, as identified by the research participants, and how it has led to lower sugarcane crop productions, the problems coming together as the ‘perfect storm’.

### Trade-Related Problems of the Fiji Sugar Industry

The study identified loss of market access, end of FairTrade (FT) premium, fluctuating market prices, and geographical isolation of Fiji from the rest of the world, especially critical international sugar markets such as the UK and the EU, as some significant trade-related problems of the Fiji sugar industry. These are discussed in turn below.

### Loss of Market Access

In the aftermath of EUs withdrawal of preferential access for ACP countries, Fiji was provided with a transition period from 2012 to September 2017 (Chaudhary 2015c). After that, the EU opened its markets to all ACP products, and guaranteed prices had phased out with ACP producers treated like European producers. This switch by the EU provided the ACP member countries free access to the markets in EU. However, the EU had also been careful of not destabilising their market with exports from the ACP countries.

The loss of international EU and UK markets will indeed create many issues for us here in Fiji. It will have multiplier and ripple effects for us as a country and our people. We are one of the smallest producers of sugarcane in the world compared to countries such as Australia and Brazil…we have many populations that rely and survive on the sugar industry. A time will come when we have to increase the price of sugar for local markets also. First, we need to see how we can recuperate this industry, and then we also must solve many challenges and difficulties that we are facing. Many systemic, structural, and institutional issues need to be addressed (SCGC 2015).

Overall, this move by the EU has profound implications for the world market price of sugar, and the Fijian sugar industry, particularly the sugarcane farmers (Chaudhary 2015d). This added to the existing pressure on the industry and FSC had to borrow money to maintain the FJ$80 per tonne price to farmers, and over the years, it has become increasingly difficult to do this.

The FSC has been operating at a loss for years, and there are many reasons for it. We are trying our level best to turn the tide. We need the farmers to understand that the quality of the sugar produced makes the difference in the price, not the sugarcane tonnage. One can be involved in high sugarcane productions, but if that harvest does not give us the quality of sugar we require, we will be making losses as millers. It means that the consumers demand high quality of sugar, and if we are not producing high sugar qualities, then we are no match to those countries doing so. In addition, a significant issue now is that we will have to see how we pay our farmers. It is because we will no longer have the luxury of three times the regular price of sugar paid to us, the FT is pulling out, and at the same time seeing that we remain viable as the miller, and that is why we continue to re-iterate and re-emphasise the need for quality sugarcane cultivation and production (FSC 2015).

### End of FT Premium

Fiji has also since 2010 been receiving an FT premium for its sugar, with the FT certification of sugar in Fiji that started with the farmers cultivating sugarcane on the island of Vanua Levu. The FT has been an alternative approach to conventional trade and was based on a partnership between producers and consumers. The FT aimed to empower farmers and workers in Fiji through improved terms of trade that will help advance the working and living conditions of the farmers and their communities.

A report by the Secretariat of the Pacific Community (SPC) in 2012 found that FT certification produced significant economic benefits for the farmers. The report estimated that the economic impact of FT certification of all farmers serving the Labasa sugar mill in Vanua Levu amounted to FJ$9,094,473, considering all costs and benefits and assuming the benefits and costs were incurred over 12 years (Island Business 2012). Such a partnership helped incentivise the system and compelled the farmers to engage in higher crop production rates as it was a win–win situation for the farmers, the millers, and the FT in meeting its mandates.

According to Island Business (2012), FT partnership and certifications represented a return of FJ$6.48 for every dollar spent on gaining certification, including those paid by farmers and donors—the EU, the principal contributor together with the SPC. The subsequent extension of FT certification to other sugarcane-producing areas in the West of Viti Levu raked in several million-dollar worth of additional income benefits to the sugarcane farmers at relatively low cost. Moreover, it provided associated benefits for the sugarcane farmers for many years. However, in 2015 the FT announced that from 2016 they would no longer be paying premiums from the sale of sugar. This resulted in the immediate loss of some FJ$13 million worth of income from traditional sugar buyers, Tate & Lyle Company of UK (Chaudhary 2015c).

It is quite unfortunate that the FT has decided to leave. We will now be losing many incentives that were once available to us. This decision by the FT will impact crop production, specifically for the smallholder farmers. Apart from this, there will be some setbacks to future community development projects in the sugarcane fields. We do not know if there will ever be a replacement as the FT (Labasa Farmer 2015).

The ending of the FT premiums has also seen sugarcane farmer communities facing serious setbacks that have directly hindered their community development projects, which has benefitted families in the sugarcane growing areas. The FJ$25 million premia that have been received over the past four years since 2011 from the sales of sugar registered under FT products have assisted the farmers by reducing their costs through subsidies on farming equipment, improving drainage systems, and purchasing fertilisers. The decision by Tate & Lyle to withdraw from their commitments to buy 100,000 tonnes of sugar at FT premium directly affected the FSC, who now had to search for other alternative markets to sell the FT certified sugars.

### Fluctuating Market Prices

With the withdrawal of preferential EU quota access and FT premiums, Fiji has become heavily dependent upon world market prices for sugar, which fluctuates annually. Although these annual fluctuations are a significant concern for the sugar industry, specifically for the FSC, the price received for out-of-quota sugar tends to follow the world market price.

See, there is always an annual forecast for world sugar price, but this fluctuates also. The farmers will need to understand this. So, whatever the country receives at the global levels, they can only work from there to pay the prices to the farmers. The farmers have been receiving decent amounts for their produce. On top of that, the farmers also receive so many different types of subsidies from the Government. So, when one considers all these, the actual amount paid out to farmers in Fiji has always been more than FJ$100.00 per tonne (CPA 2015).

Between 2011 and 2015, Fiji tried its best to attain the world sugar price, when the price paid to the farmers locally deteriorated. The deterioration of sugar prices placed local farmers at a disadvantage, which escalated the already boiling tensions between the FSC and the farmers. As a result, the government contributed additional payments to the farmers apart from what was paid to them by the FSC. Thus, to some degree, it helped regain farmer confidence, but only for a short time.

### Geographical Isolation

**Fig. 2:**
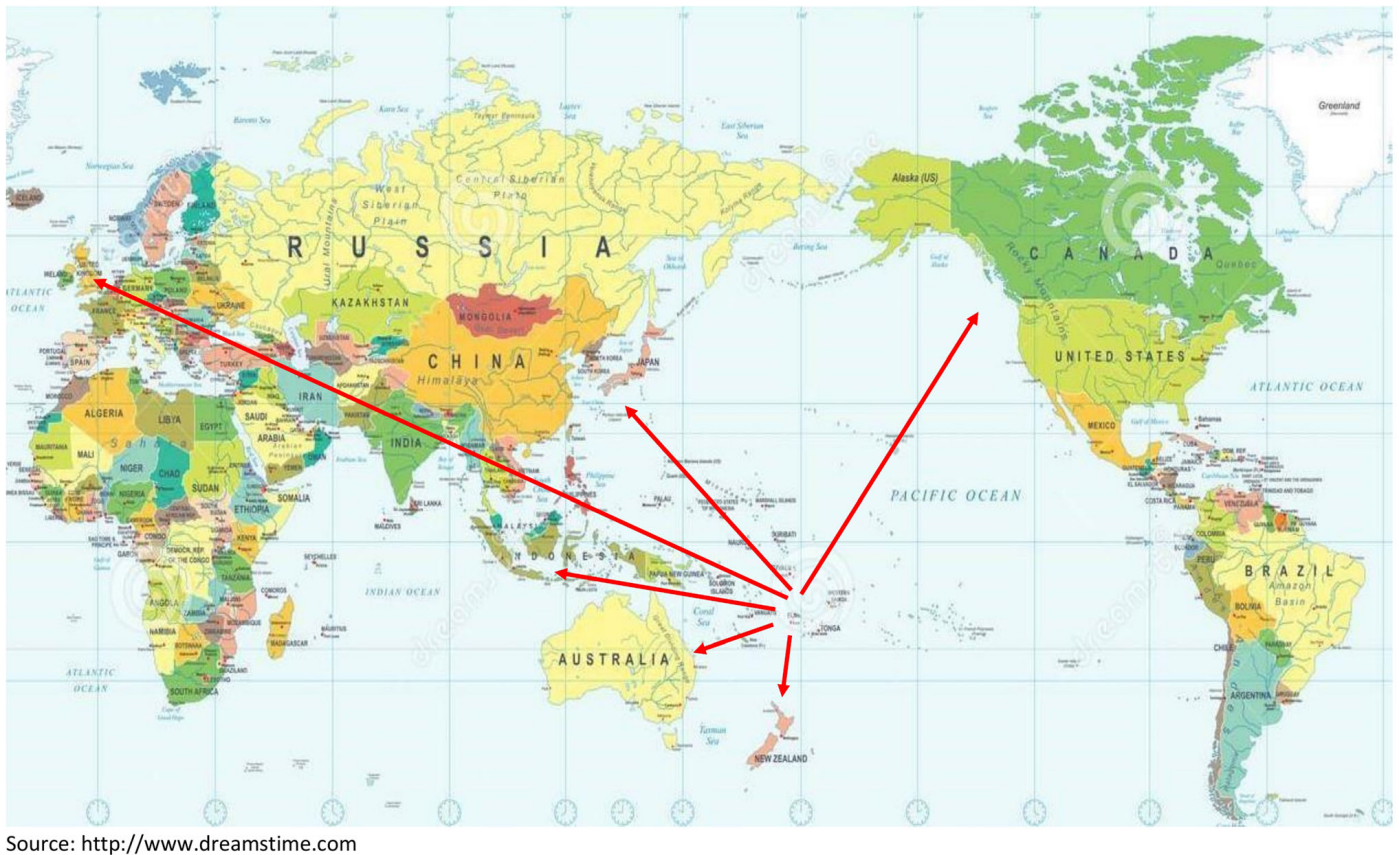
Map showing Fiji’s distance to major international sugar markets

Fiji, like other Pacific small island developing nations, is a victim of the oppression of distance. Small domestic markets and the country’s remoteness are Fiji’s most prominent barriers to economic development and success. In addition, an enormous expanse of ocean separates and isolates Fiji from the rest of the world, and high transportation costs tend to restrain trade within the region and international markets (Fig. 2).

For Fiji, being the only sugar-producing nation in the South Pacific, isolation has severe implications for its sugar industry and the farmers. The global oil market volatility further aggravates such issues. A hike in oil price often triggers an increase in the cost of fuel for farm machinery, such as tractors to work the farms and generators to produce household electricity for those farmers living in interiors of the sugarcane-producing areas and do not have access to utilities.

### Macro- and Micro-Problems of the Fiji Sugar Industry

The sugar industry has also been facing a growing number of macro- and micro-problems. These are declining sugarcane production, declining numbers of farmers, loss of farmer confidence, loss of productive land to urban development, milling inefficiency, climate, climate change, and pests and diseases. These are briefly discussed below.

### Declining Production

**Fig. 3:**
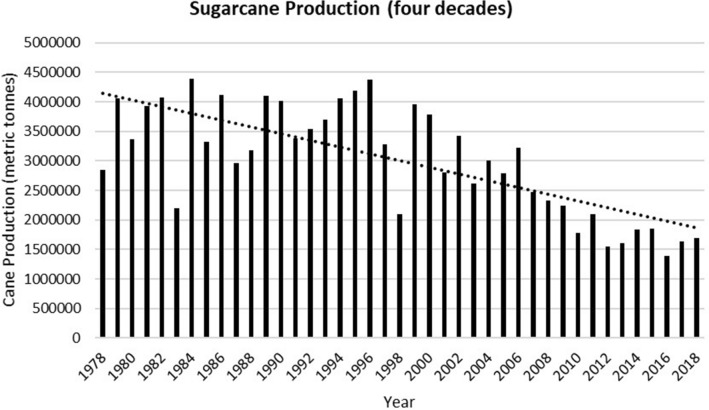
Trend for sugarcane production

**Fig. 4:**
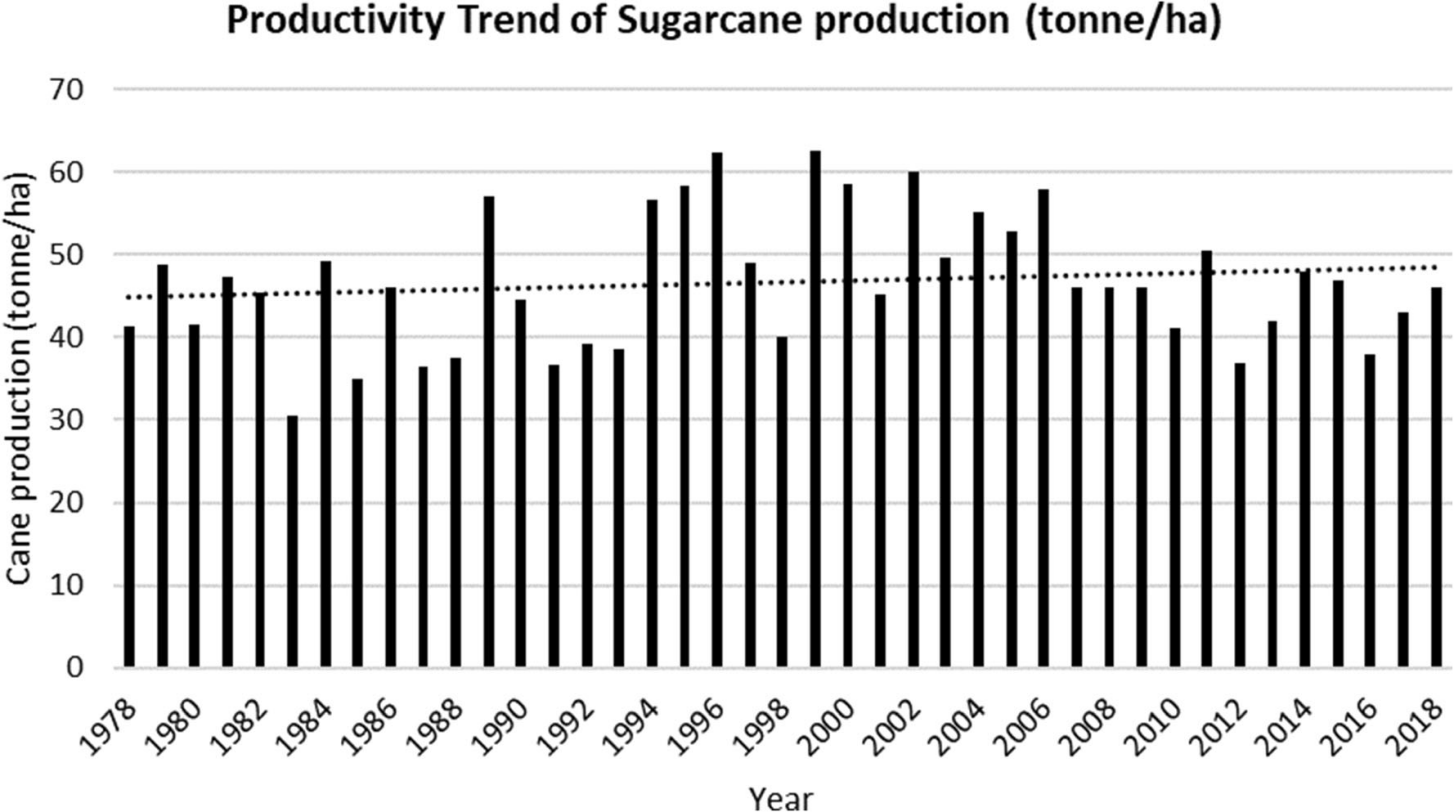
Productivity trend for sugarcane production

In the past two decades, Fiji’s sugarcane crop production has declined considerably (Figs. 3 and 4). It has contracted by more than half, from 4.06 million metric tonnes of sugarcane crushed in 1994 to 1.83 million metric tonnes in 2014 (SCGC 2016), to 1.6 million metric tonnes in 2018 (FSC 2019). Similarly, the number of registered farmers has also declined. For example, in 1994, there were 22,807 registered farmers. By 2018, their numbers had declined to 16,666, out of which only 11,902 were active farmers (FSC 2019).

The total area under sugarcane production has also contracted by almost half, from 74,388 hectares in 1994 to 38,248 hectares in 2013 (FSC 2014). Thus, while there was an upward trend in the area under cultivation before 1990, it reduced significantly thereafter. This trend is partly attributed to the non-renewal of land leases under sugarcane crop production by the native *iTaukei* landowners, resulting in a decline in the number of *Girmitiya* sugarcane farmers, when their farm leases began to expire in 1997. As a result of nothing being done to assist these farmers in renewing their farm leases, many farmers started to lose confidence in the sugar industry and started to leave the sugarcane fields, which further resulted in the dwindling of the industry.

For anyone, confidence and motivation are essential to continue to be productive. The same can be said for sugarcane farmers. However, if we continue to face significant issues such as the expiry of land tenure, many farmers will not want to continue farming and producing crops. We also have short term and long terms goals and plans for our families and crop production. We can only commit to producing better and more once such problems have been solved amicably (Rakiraki Farmer 2015).

### Declining Numbers of Farmers

**Fig. 5:**
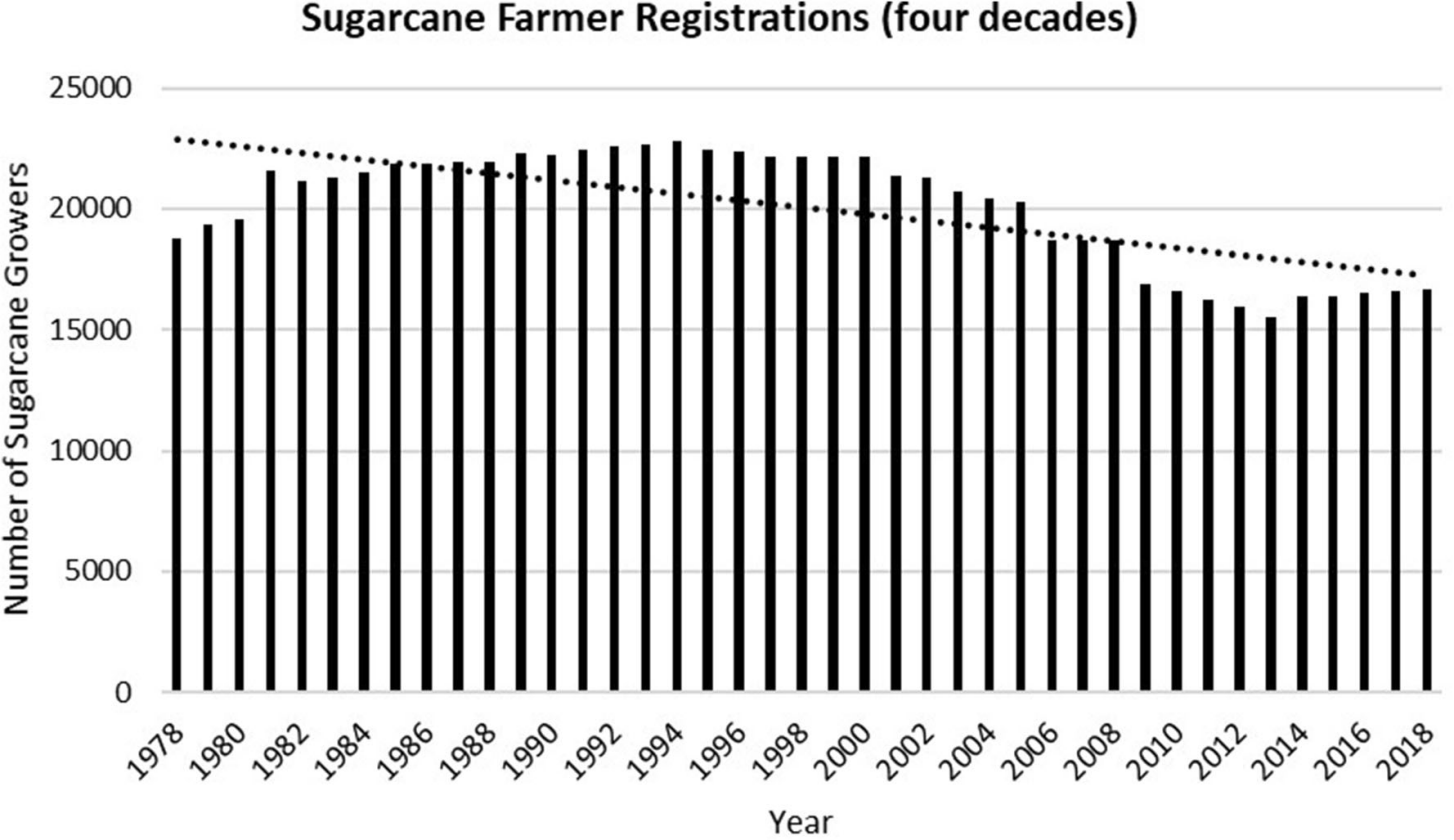
Trend for sugarcane farmer registrations

A significant challenge in Fiji is that majority of the people do not find agriculture a lucrative and attractive occupation. Specifically, the young generations find agriculture and sugarcane farming unrewarding, time and energy consuming work, resulting in a negative demographic shift (Fig. 5).

Another major challenge is that we cannot entice enough the young generations to stay behind in the sugarcane growing areas and take up sugarcane farming as an occupation like their parents and grandparents. This is because the current pool of young generations in Fiji do not see agriculture as an occupation or business, they want to invest in. They would rather work towards a white-collar job (MoA 2015).

**Fig. 6:**
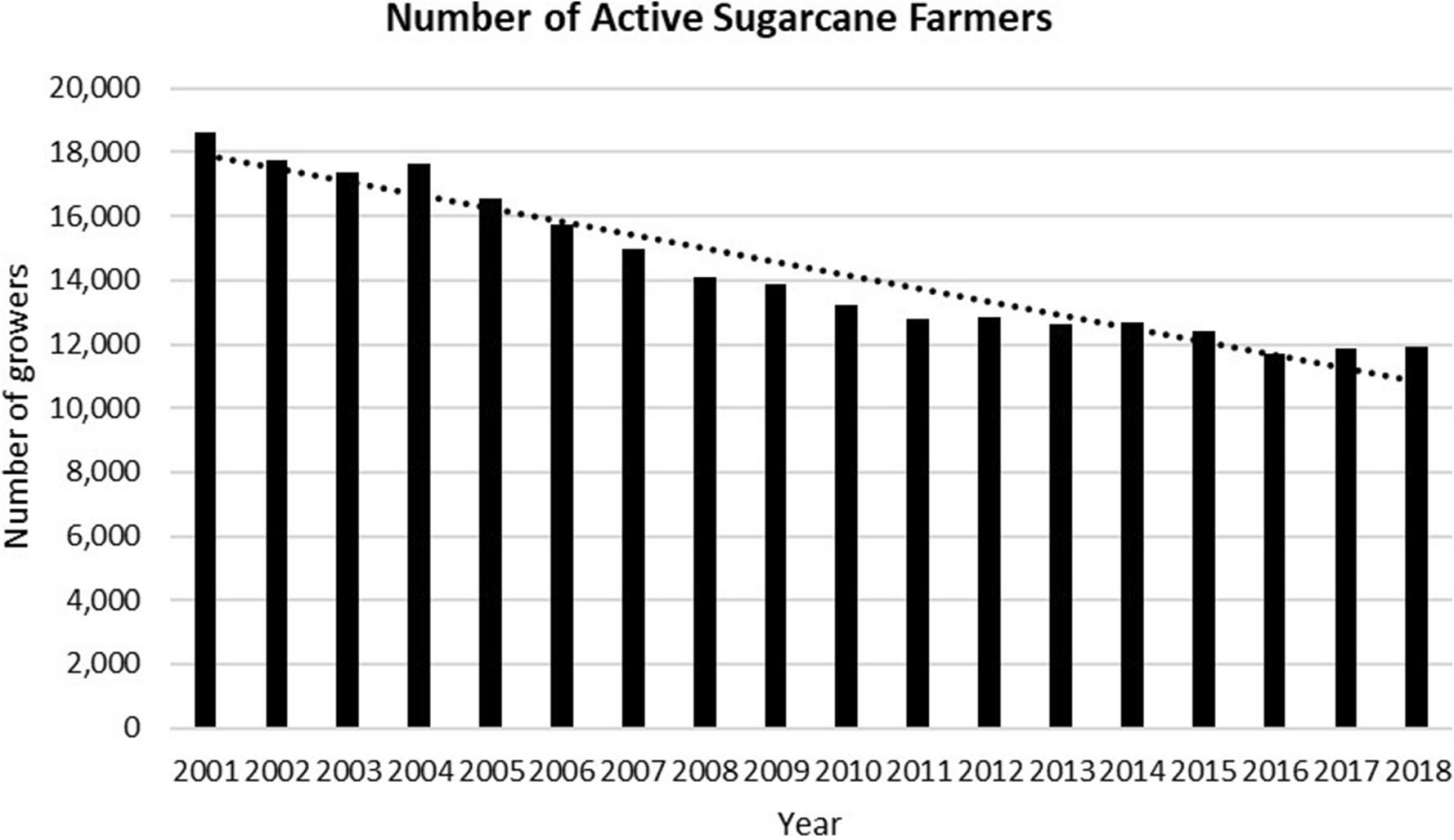
Trend showing active farmers (Years 2001–2011)

Consequently, the sugar industry is now also challenged by a growing number of farmers who have started to treat sugarcane farming as a hobby. These are ‘hobby farmers’ who do not take sugarcane farming seriously but continue to retain sugarcane farms for various reasons: historical attachment to the sugar industry or sentimental values tied to sugarcane farming. Many farmers continue to plant because they still have the land available to them, and once their leases expire, they may or will take up other occupations elsewhere (Fig. 6). It has been the case of many farmers who left sugarcane production when land for sugarcane cultivation became unavailable for one reason or another.

The hobby farmers also have other sources of income such as investments in taxi operations, or they work in supermarkets, garages, or their sons and daughters are working in professional capacities as teachers, doctors, and nurses. In addition, they also have children living overseas who send them remittances. Therefore, these hobby farmers remain unconcerned about increasing their crop productions, and they have other forms of income to fall back on. Such dynamics are highly concerning for an already subsiding sugar industry, where 80% of sugarcane farmers are now mostly hobby farmers and do not take sugarcane farming as a business.

If one goes around the country where sugarcane farmers live, they will see that they are not entirely dependent on sugarcane production for their livelihoods. At least one member from a farmer household is living elsewhere and are engaged in different income-generating activities or employment. Moreover, many households are also taking up other activities to generate additional incomes, so sugarcane farming is turning into a side business, more like something good to have but not a strategic investment (SCGC 2015).

### Loss of Farmer Confidence

The loss of confidence in the sugar industry is connected to farmer struggles, relating to expenses on the farm and costs associated with harvesting and cartage of sugarcane crops to the mills that always take a higher toll on their incomes. For many smallholder sugarcane farmers, these costs at times tend to be higher than what they receive from the sale of their sugarcane crops to the FSC. In other words, most of them have been making losses or remain at break-even points.

We end up paying for so many expenses. We must pay the sugarcane cutters; we must pay the high transportation costs of the harvested produce to the mills. Apart from this, we also have household expenditures. Many a time, the costs associated with sugarcane farming tends to be higher than what we receive as income (Sigatoka Farmer 2015).

On the other hand, the FSC's position is that the income farmers receive for their sale of sugarcane to the FSC is also affected by shipping costs and schedules, molasses prices, and the negative currency movements, apart from volatile global markets. As a result, many farmers have already diversified their sugarcane fields to include other forms of agriculture for commercial and subsistence purposes (Dean 2019).

### Loss of Productive Land to Urban Development

During the colonial era, most of the sugarcane-producing areas in Fiji were concentrated close to the mills and the railway lines. However, in the past two decades, substantial amounts of land have been lost to expanding and developing towns, cities, and residential areas. For example, the prime flat lands having higher production potential than marginal land (hilly terrains, marshy or waterlogged soils) have mostly been taken up for development purposes. As a result, approximately 20–25 thousand hectares of productive fallow land reserved for sugarcane farming has been lost, contributing to lower crop productions. Currently, the flat areas remaining under production of sugarcane crops stand at 27% only. The rest of the sugarcane harvests comes from marginal areas.

Expansion and development meant that those who took up sugarcane farming had no option but were pushed to marginal lands to grow their crops. Consequently, this has proved to be disadvantageous in the long term, in terms of lower crop productions and the use of high amounts of fertilisers and machinery to grow sugarcane crops. Most of these marginal lands, for example, the hilly terrains and slopy farms, require a lot of labour and machinery to grow the crops, to the extent that when the harvest time comes, and it is rainy weather, we have to stop harvesting because no lorry drivers want to drive their lorries in these hilly and slopy terrains as the risks are high for them (Sigatoka Farmer 2015).

In addition, the expansion of farms inland, has increased the distance from farm to the mills considerably. Consequently, transportation and carting have become a significant problem, increasing cartage costs for the farmers because of failure on FSC's part to manage the railway system well for transporting the harvests.

### Milling Inefficiency

Over the years, the FSC has also failed to consistently produce sugar more than the preferential quota, as mill breakdowns were frequent whenever mill throughput was increased. Moreover, whether planned or unplanned, mill stoppages have seen FSC facing huge criticisms by the farmers for the lack of skills and management expertise. Similarly, the rail networks’ deteriorated conditions and the mills’ inability to handle increased sugarcane volumes have seen crushing seasons being extended (Chaudhary 2015a).

Poorly maintained rail networks increase the time for the harvested sugarcanes to reach the mill, while other issues such as the malfunctioning of the mill’s generator led to the stopping of the crushing altogether. Likewise, mill crushing machinery at times have become non-functional in the process of crushing, requiring maintenance work. During such events, harvested sugarcane produce remains stuck for days at the mills and at the farms, which eventually deteriorates the quality of the harvest.

The FSC is quick to blame the farmers for not producing quality crops. However, at the same time, they should realise that even their milling capabilities lack quality. Most of the time, they stop milling because of their old technology or because their mills have not been well maintained. They tend to blame the farmers for our shortcomings continuously, yet have they ever tried to look at the shortcoming on their end (Tavua Farmer 2015).

Some other issues remaining unsolved at the mills during the research period in 2015 were the non-availability of proper sanitation and toilet facilities and the prolonged waiting time that lorry drivers must face every day during the crushing seasons (Naidu 2015). The drivers must wait in the queue for as long as 15 to 20 hours before dumping the loaded harvests and returning. The waiting time results in the loss of productive work hours and increases health risks for the drivers.

### Climatic Condition, Climate Change, and Pests and Diseases

Fiji sits in an area in the Pacific that is always prone to natural disasters. For many years, increasing frequencies of hurricanes and storms coupled with flooding continue to impact the sugar industry. The country has also experienced two years of continuous droughts and four to six devastating cyclones since 2010. Also, crops such as sugarcane are threatened by many plant diseases and pests because of climatic disturbances.

#### Drought

Drought has significant impacts on the farmers, their crops and their livestock. Farmers usually find themselves caught with uphill battles in maintaining their crops and livestock during drought. The farmers keep livestock such as bullocks and horses for several reasons. The livestock is used for animal traction during the cultivation of various crops including sugarcane, as a food source, bartering in exchange for goods and services, and selling them for income generation whenever required.

During the dry seasons, farmers must spend money to purchase alternative food supplies such as copra and mill-mix to feed their animals because of the lack of availability of green pastures. In addition, some farmers are compelled to travel long distances either by foot or by some other means to fetch drinking water for their livestock, in addition to fetching water for sugarcane and other crops. Some farmers also face cattle invasions during dry spells, whereby livestock invade the farms to feed on the sugarcane and other vital crops. Often, this leads to the new plants being uprooted. Also, once the crop has been fed upon, it is difficult to recover them. As a result, farmers suffer considerable crop losses from livestock invasion.

#### Cyclones

Many of the cyclones in Fiji have caused widespread destruction to the farmers’ properties, the sugarcane fields, and sugar industry infrastructure. The cyclones result in extensive damage to the farmers, who already struggle on low incomes from the sugarcane they harvest. They disrupt economic activity. Many farmers in Fiji have also lost their livelihoods, shelter, and belongings because of naturally occurring catastrophic events that usually pound the country.

For example, the severe Category 5 Cyclone Winston in 2016 caused substantial damages to the sugar industry (Radio New Zealand 2015). With wind gusts of up to 330 kms per hour, it had flattened the farms and uprooted the sugarcane crops. Many of the farms also remained waterlogged for several days. A preliminary assessment of damages to sugarcane farms and infrastructure caused by Winston was pegged at US$83 million. It also caused an 80% loss of the total sugarcane crops, with Tavua-Rakiraki sugarcane-producing areas on the island of Viti Levu being worst affected, and their Penang Mill in Rakiraki shutting down and FSC declaring it non-operational after the cyclone (Chaudhary 2016).

#### Climate Change

Climate Change remains an existential threat to Fiji and the sugar industry. Sugarcane needs good rain, the right amount of sunshine and moisture for its growth. However, the weather patterns have deviated substantially, which affects the crops growth. Another effect of climate change, the rising sea levels, has long ago started to intrude into the sugarcane fields because of broken floodgates. Likewise, the sugarcane rail lines continue to be corroded by the intrusion of seawater onto land. As a result, the industry will need to invest significant money in re-routing and remounting these corroding rail lines.

#### Pests, Feral Animals and Disease

The infestation of pests and diseases is expected in the sugarcane fields if they are not appropriately managed. Common pests in the sugarcane fields include weeds such as grasses, broadleaf and creepers, sugarcane weevil borer (*Rhabdoscelus obscurus*), rodents, mongoose, feral pigs, unsupervised cattle, ants, and termites. The weeds are responsible for up to 25% reduction in sugarcane yields. The sugarcane crops are also exposed to diseases such as Fiji Leaf Gall Disease (FLGD) and Ratoon Stunting Diseases (RSD). Occurrences of these diseases are common. Both diseases have been found to have a devastating effect, both on ratoon and the newly planted crops. The FLGD results in losses of 100% in sugarcane yield and is more common in susceptible sugarcane varieties such as the *Mana* variety, while for the RSD the incidence rate is estimated at 28%.

The FLGD is responsible for stunting sugarcane plants, causing raised whitish yellow swelling (galls) on the backside of the leaf blade and midrib, with leaf tops showing ‘bitten off’ symptoms. RSD is responsible for slowing the germination of the plant, affecting its health, and reducing the number of stalks, leaving the crop with short, thinner, and stunted leaf growth. With RSD, the nodes of the matured sugarcanes usually also suffer from discolouration in their vascular bundles. Although smut disease is not prevalent in the country yet, the SRIF recommends that Fiji prepare itself for this disease. The smut disease has spread around the globe in the 1970s and 1980s. It has been responsible for 30–100% loss of sugarcane crop production. In the past ten years, the disease has managed to reach Australia. Papua New Guinea and Fiji are the only two sugar-producing nations devoid of the smut disease.

### Farmer Problems

Some problems facing the sugar industry in Fiji are unique to the sugarcane farmers, and for this reason, they are dealt with separately in this study, as opposed to grouping them into macro- and micro-problems of the sugar industry. These problems are as follows: industry representation, payment struggles, security of land tenure, scarcity of labour, rising costs, and internal politics related to sugarcane farming.

### Industry Representation

The sugar industry in Fiji consists of many stakeholders (Table 1). The sugarcane farmers are the largest stakeholder in the Fijian sugar industry, but they have a weak say in the industry (Chaudhary 2015b). There is evidence that the sugarcane farmers have constantly been searching for a more prominent voice. For the farmers, they feel that decisions are being made in air-conditioned offices without taking account of the fundamental issues on the ground. During the research, depressing statements from the farmers continued to surface, demonstrating that the farmers were fighting a losing battle. The farmers feel that decision making is not consultative, and solutions are implemented without seeking their views and advice on how best to address them.

It is just like a soccer field. Farmers are the leading players, and we have ball in our hands. There is no one asking us as for how we are to play the game with this ball…the linesmen…the referee and the coach [referring to the key institutional stakeholders of the industry such as the FSC, SRIF, and others] do not know what is about to happen and how the players are to play the game. We feel that they are not asking us because we do not have any legal representation in the industry. We need someone to speak on our behalf. There is only an administration part to the Growers Council left, and the council has their Board missing. The Government should listen to the farmer issues, and if they do not want to, they are not obliged (Labasa Farmer 2015).

### Sugarcane Payment Struggles

For the farmers, the sugarcane payment formula set out by the Master Awards—the governing legislation of the sugar industry for the distribution of incomes generated through the sale of sugar by the FSC to the sugarcane farmers works in favour of increasing the sugarcane production levels without taking into consideration the conditions under which the sugarcane crop production takes place. The Master Award Clause 20.2 states that the farmers share of the sale proceeds shall be calculated in the manner set out in the Award (Table 2).

**Table 2:** Percentage proceeds of the sale of sugar

Total sugar produced	Growers’ share	The corporation’s share
Up to 325,000 tonnes	70.0%	30.0%
For every tonne over 325,000 tonnes up to 350,000 tonnes	72.5%	27.5%
For every tonne over 350,000 tonnes	75.5%	25.0%
These percentages are based on the net proceeds of sale		

In addition, having a system devoid of penalties, especially for crop production more than the ‘basic farm allotment’, had encouraged production beyond the actual allocations even when the price of sugar remained low. Thus, the ‘farm allotment’ element has been beneficial for some farmers, while for others, counterproductive. In a similar vein, the preferential quota income formed a shared pool to which every farmer had access, depending on their farm output. So, for those farmers who were able to produce more than their allotment, they would have benefitted more without any penalty. In this way, the farm allotment system remained disconcerted and weak in its implementation. In addition, the method and process of payments as set out by the Master Award has also proved to be disadvantageous, majorly to the smallholders. For example, one harvesting season’s payment is spread over two consecutive sugarcane seasons and distributed in percentages from the sales proceeds into 3–5 payouts.

### Security of Land Tenure

Another significant issue is the land tenure security. The problem with the land leasing system for sugarcane production in Fiji is related to the lease term period provided under the Agricultural Landlord Tenants Act (1976) (ALTA) legislation. Firstly, the ALTA (1976) provides a thirty-year lease period, which is one generation long. Secondly, the option of renewal rests entirely with the landlord, which inevitably puts the tenants at the mercy of their landlords when they seek land lease renewals. However, land tenure is not an issue for that small pool of sugarcane farmers with legal land title ownership in their names.

Imagine, to maintain land leases, the farmers have to make many other sacrifices. Apart from paying the regular rentals annually, we make other payments to the landowners as goodwill. They come and ask for it, and we have no option but to make such additional payments directly to them. Otherwise, they will not renew the land leases after expiry. Even after giving them the money, we are still not guaranteed that they will support lease extensions (Lautoka Farmer 2015).

This expiration of land leases, in the process, has caused a substantial loss of human resources (household labour) on the farms. In addition, the movement of people from the farms has resulted in the loss of labour for cultivation, harvesting, and transportation of the crops, most of which the farmer and his family would do themselves (FSC 2016). In their paper, Lal et al. (2001) pointed out that the commercial future of the industry is heavily dependent on the resolution of the land tenure system that has been in place since 1909 when the British colonial Government froze land ownership titles to protect indigenous property owners.

### Scarcity of Labour

**Fig. 7:**
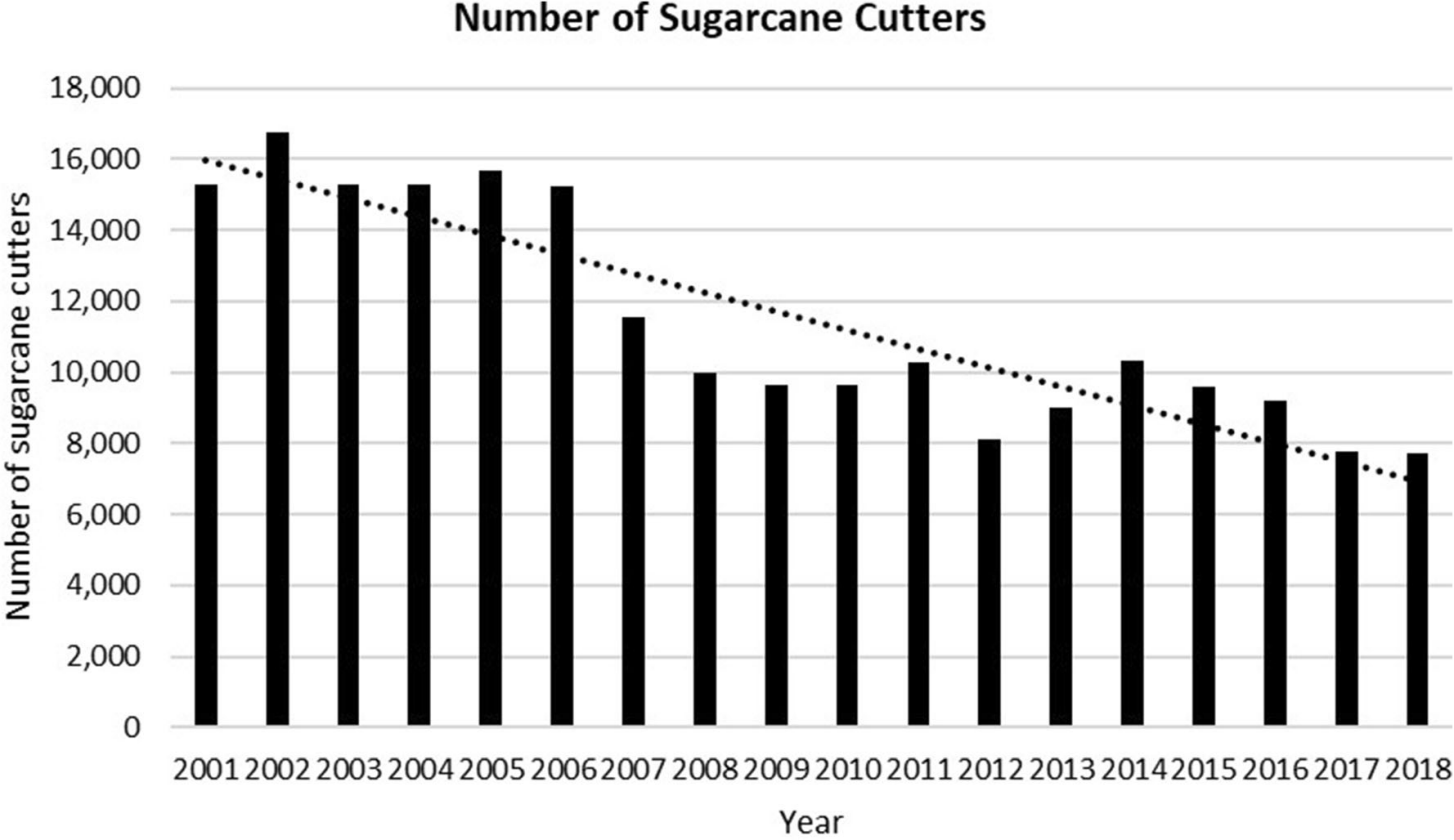
Trend showing available sugarcane cutters (Years 2001–2018)

Another primary concern for farmers is the lack of and, to some extent, the non-availability of labour to assist with harvesting (Fig. 7). While most of the current pool of farmers’ children do not want to work on the farms as they used to do twenty to thirty years ago, people also see those engaged in such agricultural occupations as third-class citizens. Having children on the farms meant that labour was readily available whenever needed. The other problem is the age factor. Once the old farmer, who is regarded as the head of the household dies, his children often do not carry on the farming legacy, and his wife may also sell the farm. At other times, the farms just sit idle unproductive.

The farmers are dependent on hiring labourers from outside their regular gang or nearby villages or sourcing people from outer islands primarily of *iTaukei* descent. Some farmers feel that these labourers do not feel any loyalty to the sugar industry. Most of these labourers are not trained and are not used to doing things on time.

Over the past decade, sugarcane cutter demands have risen considerably, for example their demands include remuneration and other benefits such as providing them with accommodation and grocery allowances if they are live-in labourers, providing tea or juice while they work in the field, three meals per day, and paying for their transportation costs, in addition to their regular wages. Other labour recruitment conditions may include making lump sum payments five to seven months before the harvesting season to bind the already limited pool of labourers so that other farmers cannot pull and utilise them in their farms. If, for example, the labourers demand upfront payment of FJ$500 per person, and if the farmer requires three individuals to help with the harvesting, that amounts to FJ$1500.00, even though it can later be deducted from their wages. The farmer is then burdened with the challenge of receiving the payments from FSC for his harvested produce in a timely and consistent manner to compensate the labourers in time.

### Rising Costs

The cost of production of sugarcane in Fiji remains exceptionally high. Despite numerous measures, for example planting of high-valued sugarcane varieties and Government subsidies being put in place, the country has failed to curbing costs. Harvesting and transportation of sugarcane crops alone takes a higher proportion and is a significant issue for the farmers and the industry. Approximately 50% of the total cost of producing one tonne of the sugarcane is attributable to harvesting and transportation alone, compared to other significant costs such as quality sugarcane seed, weedicide, pesticide, and fertilisers. The FSC has not been able to manage the rail systems well. The locomotives, rail trucks, and tram lines are almost 100 years old now, just like their milling technologies.

We have no options and also no feasible solutions that fit our context. Most of our farms are on hilly terrains, so we have to carry out manual sugarcane harvesting, and because we live far away from the mills, we have to use other forms of transport to cart our produce to the mills. Just these two activities are exorbitant (Rakiraki Farmer 2015).

Farms located outside the 20 km radius of the mills are at a disadvantage as the rail systems far away from the mills remain non-operational and cannot be accessed to transport sugarcane harvests to the mills. Therefore, those farmers living and farming within the 20 km radius of the mills can save on the high costs of transportation and are more prolific when compared to those farmers living far away from the mills.

Hence, many of the farmers have been additionally compelled to use trucks and tractors for transportation of the produce and incur additional costs. Sugarcane transportation is a heavy burden on the farmers, although the FSC subsidises it through grant schemes. The late repairs to sugarcane access roads have impacted the industry immensely, with disruptions to harvesting and transportation for many years.

Even when the transportation costs are subsidised, we have to ensure that the transportation for harvested sugarcanes are available in time. If we do not secure the lorry drivers and their trucks on time, our harvesting rhythm is affected, affecting the overall harvesting of the produce. Also, fuel costs tend to increase now and then, so the sugarcane transport operators charge us accordingly. Imagine those farmers who live at extremely far distances to the mills, like the interiors. Even the sugarcane access roads are not well maintained on time (Seaqaqa Farmer 2015).

The high operation of trucks also impact the road systems during peak seasons of harvesting and crushing, when the roads become heavily congested with trucks and lorries transporting the harvested sugarcane. Furthermore, the cost of inputs such as weedicides, pesticides, and fertilisers has skyrocketed, impacting the income and livelihood of the farmers.

### Internal Politics

Sugarcane farming in Fiji is constantly being transformed and reshaped by changing relationships within the communities. Any discussion of internal politics in sugarcane farming must consider differences in settlement types, worldviews, trade relations, and socio-economic factors. Despite the appearance of healthy relationships within rural farming communities in Fiji, conflicts and status rivalries do exist within the farmers. Some of these problems and their sources are discussed below.

#### Farmer Inequalities

The farmers feel that FSC is largely to be blamed for most of the sugar industry’s problems. The FSC is seen as a domineering monopolistic miller that has created rivalries among the smallholder sugarcane farmers and encouraged some farmers to expand their sugarcane crop productions beyond their ‘farm basic allotment’ (cf. Chand 2005). Moreover, they find FSC reluctant to hold farmers accountable, especially those who produce more than their essential farm allotment quotas. This is another way how the farmers perceive farm allotments to be working to their disadvantage.

For the farmers, they argue that there is no threshold to production levels, and this is probably one reason why farmers generally differ in terms of their measured output if farm size, soil characteristics, and climatic conditions were to be kept consistent. It also means that when penalties are not applied, farmers can start to produce more than their ‘farm basic allotments’ or also be caught amid failing to produce the required production levels without being held accountable. Similarly, not all farm settings are equal; one farm varies from another temporally and spatially. In addition, favourable natural settings and informal rural political relationships tend to impact sugarcane crop cultivation, production, and yield.

Given the above scenario, one farm’s output more than the typical ‘farm basic allotment’ would help offset that of another, but the system also allows the ‘lucky’ farmer to earn super-normal profits while the more marginalised ones are further marginalised. The politics worsen this situation at the farmer gang level (a collective group of farmers in one area), when prominent farmers are favoured over others in the same gang, or when prominent sectors are favoured over the other sectors in a milling area. The output level may also be affected by unforeseen circumstances and the prevalence of favourable or unfavourable weather conditions in the different parts of the country where sugarcane is grown.

#### Farmer Rivalries

Other farmer issues have to do with farmer rivalries, the most common related to sugarcane field fires. There have been an excessive number of cases of fire destroying the sugarcane fields reported by farmers every season. Most of these reports are related to cases of arson that are very common in the sugarcane fields and are majorly due to political and social tensions among farmers or the extended families of the farmers, often a result of fights, jealousy, or hatred. At times, the sugarcane fields are intentionally lit because the farmer wants to hasten the process of harvesting and crushing, although it is illegal to do so. Other times, the reckless behaviour of individuals has led to fires, for example, lit cigarettes thrown in the fields. Unfortunately, at the time of the research, there have been no policies or arrangements institutionalised or initiated to get the sugarcane farms insured. Therefore, if an accident or a disaster occurs due to unforeseen circumstances, the farmer would not be compensated.

I have had my sugarcane farms lit up. It is only because maybe I have had issues with my neighbouring farmers. I have been a hard-working farmer throughout my life, so has been my ancestors. When we start to produce more crops than others, they tend to eye us, and when they find the opportunity, they light our farms. It is only because of competition. Some other problems are community-related, for example, when one may not be accepted by the community in which they are living. Other social ills and contentions arise because of religion and many other issues (Nadi Farmer 2015).

#### Favouritism and Power

Apart from the problems mentioned, farmers also face favouritism by the FSC field officers, who tend to favour prominent and well-established farmers. Some smallholder farmers also noted the issue of bribery. They state that large holding sugarcane farmers can bribe the officers with both cash and kind so that they can gain approval to harvest their sugarcane crops much earlier than the rest when the crushing season begins. In addition, a small farmer in a gang will be a lesser priority than the others and will have a lesser say in decision-making.

In my gang, I consider myself to be the least privileged one. I am quiet, and I am powerless because I do not have many possessions like other farmers. What I am trying to say is that you must have more possessions over others, be influential, and have the right connections in the industry. I have not been able to make my presence felt, unfortunately (Labasa Farmer 2015).

Further, smaller farmers are also unable to secure labourers and become dependent on the more prominent farmers from their gang or from other sectors, who may assist these unfortunate farmers once their sugarcane crops have been harvested. Moreover, the weaker farmers also face discrimination from FSC personnel. For example, if FSC staff have stated that they will deliver fertiliser or implements to a farmer on a specific date, and it does not arrive, these weaker farmers can only act or follow up with the FSC personnel after three or four days. However, for some farmers, such as those that are more powerful and influential, there is no delay, and everything seems to run smoothly, suggesting that the sugar industry in Fiji is also plagued by inequality and exclusivity. This unequal and exclusive treatment is also responsible for lower crop productions by the farmers as it affects their morale and confidence in the industry.

### Strategies to Improve the Sugar Industry: A Snapshot

The Fijian sugar industry needs drastic changes to its trade mechanisms and strategies and those related to macro- and micro-level challenges, especially measures to improve the sugarcane production, both in terms of quality and quantity. Interventions relating to the introduction of new technologies, new farming methods combined with the traditional wisdom of the farmers, and the onboarding of more skilful professionals for the management of the industry are required to reverse the declining sugarcane crop production.

In addition, sugarcane farms and sugarcane-based products will need to be diversified at the local levels while ensuring that the processes associated with diversification remain environmentally sustainable. In a similar vein, specific policy interventions to boost farmer morale and confidence in the industry are urgent for the farmers to increase crop production. The stakeholders involved, specifically the miller, should consider providing a more conducive economic environment for the farmers to be motivated to produce more. They also need to consider other interventions, for example, a quality-based payment system for increasing sugarcane crop production and rational methods for determining the price of sugar—the money paid out to the farmers for their produce and all other products and by-products sold in the local markets. Additionally, measures to further improve the research and development are crucial at this stage of the sugar industry. Similarly, the governance structures of the institutional stakeholders making up the sugar industry should be reviewed to reflect the current circumstances and contexts of the industry to ensure their effectiveness and to create positive ripple effects for all actors in the system.

Some other practical interventions that the government could implement to recuperate the sugar industry can include assistance, for example, towards system bio-diversification, soil fertility conservation and management, optimisation of nutrient and energy cycles and processes, optimal use of natural and locally available resources, maintenance of high levels of resilience in terms of systems sustainability and stability, and optimal use of renewable energy in the sugarcane fields. Furthermore, emphasis will also need to be placed on community led innovation and the exchange of resources and knowledge among the farmers, permitting the co-creation of a sustainable sugarcane agricultural system. For instance, the traditional knowledge of sugarcane farmers could be an essential source of innovation, leading not only to improved farmer livelihoods but also to improved industry performance.

## Conclusion

Sugarcane is one of the most significant crops cultivated in Fiji and for over a century has shaped the development of the country’s economy. However, for the past 20 years, both sugarcane and sugar production has declined steadily, by approximately 50%. This study has critically explored and analysed the reasons that has led to gradual decline of the sugar industry in Fiji, vis-à-vis ecological, environmental, geopolitical, and socio-economic forces at play and with limited market development.

The Fijian sugar industry in the current state is facing a ‘perfect storm’. As a result, the farmers do not see a promising future and are migrating from remote and rural sugarcane-producing areas to urban centres of Fiji and elsewhere overseas in search of more secure employment and better education for their children. Such a trend is also exacerbating the loss of confidence in the industry. Furthermore, the current COVID-19 pandemic has added to these difficulties. Therefore, as discussed above, it is vital to identify some important policy and practical measures to reform the sugar industry in Fiji and boost farmer confidence and motivation as critical ingredients for increasing sugarcane crop cultivation, production, yield, and for farmers to continue in sugarcane farming business.
